# Evolutionary Consequences of Altered Atmospheric Oxygen in *Drosophila melanogaster*

**DOI:** 10.1371/journal.pone.0026876

**Published:** 2011-10-28

**Authors:** Marc Charette, Charles-A. Darveau, Steve F. Perry, Howard D. Rundle

**Affiliations:** Department of Biology, University of Ottawa, Ottawa, Ontario, Canada; Oregon Health and Science University, United States of America

## Abstract

Twelve replicate populations of *Drosophila melanogaster*, all derived from a common ancestor, were independently evolved for 34+ generations in one of three treatment environments of varying PO_2_: hypoxia (5.0–10.1 kPa), normoxia (21.3 kPa), and hyperoxia (40.5 kPa). Several traits related to whole animal performance and metabolism were assayed at various stages via “common garden” and reciprocal transplant assays to directly compare evolved and acclimatory differences among treatments. Results clearly demonstrate the evolution of a greater tolerance to acute hypoxia in the hypoxia-evolved populations, consistent with adaptation to this environment. Greater hypoxia tolerance was associated with an increase in citrate synthase activity in fly homogenate when compared to normoxic (control) populations, suggesting an increase in mitochondrial volume density in these populations. In contrast, no direct evidence of increased performance of the hyperoxia-evolved populations was detected, although a significant decrease in the tolerance of these populations to acute hypoxia suggests a cost to adaptation to hyperoxia. Hyperoxia-evolved populations had lower productivity overall (i.e., across treatment environments) and there was no evidence that hypoxia or hyperoxia-evolved populations had greatest productivity or longevity in their respective treatment environments, suggesting that these assays failed to capture the components of fitness relevant to adaptation.

## Introduction

Deviation from normal atmospheric partial pressure of O_2_ (normoxia: aPO_2_) imposes biological consequences in many aerobic organisms. Under hypoxic conditions, where aPO_2_ is reduced, aerobic metabolism is impacted and several acclimatory processes can be triggered at many levels of organization ranging from increased O_2_ uptake [1], [2] to changes in mitochondrial physiology/metabolism [3]. Conversely, hyperoxia (increased aPO_2_) can augment oxidative stress. For example, exposure to 100% aPO_2_ can overwhelm antioxidant defenses and induce damage at the mitochondrial and cellular level, with direct impacts on individual longevity [4], [5], [6]. Previous work examining the effects of altered aPO_2_ on insects has tended to focus, although not exclusively, on hypoxia and its consequences for a variety of traits, addressing phenotypic plasticity, respiratory physiology, and aerobic/anaerobic metabolism, as well as evolved responses to artificial selection for increased hypoxia tolerance [3], [7], [8], [9], [10], [11]. Studies examining the effects of hyperoxia have focused on the extent and location of oxidative damage resulting from high aPO_2_ environments, behaviours associated with preventing such damage, as well as the consequences of oxidative damage to mitochondrial physiology/phenotype [6], [12], [13]. Moreover, studies have examined if evolved responses differed from acclimatory responses with respect to respiratory physiology and longevity [14], [15].

To date, however, no study has provided an integrative view of the evolutionary consequences of hypoxia or hyperoxia, including possible fitness consequences underlying adaptation to these environments. Our understanding of the phenotypic basis of adaptation to such environments is also incomplete. The present study uses replicate populations of *Drosophila melanogaster* as a model system to characterize the evolutionary response to altered aPO_2_. This approach takes advantage of the power of this species in permitting both experimental evolution and a detailed assessment of physiological and performance traits, along with direct tests for adaptation to treatment environments.

The evolution experiment involved 12 replicate populations derived from a single common ancestor, with four populations allowed to evolve independently in one of three treatment environments consisting of altered aPO_2_ levels: hypoxia (5–10% O_2_), normoxia (21% O_2_), or hyperoxia (40% O_2_). To characterize the response to selection in these populations, we assayed an array of traits at different times during experimental evolution, always using flies raised for two generations in a common (normoxic) environment (i.e. a ‘common garden’) to remove any environmental effects and hence to isolate evolved differences. All traits were then measured under common normoxic conditions or within the context of a full reciprocal transplant among all three of the treatment environments (hypoxia, normoxia, hyperoxia). In all cases, analyses treated populations, and not individuals or groups thereof, as the independent unit of replication in tests of treatment effects to avoid pseudoreplication.

The traits assayed fell into three broad categories. The first related to whole organism performance/fitness under altered atmospheric compositions, in particular tolerance and recovery from acute hypoxia, tolerance of prolonged oxidative stress, longevity, and productivity (number of adult offspring produced by replicate male-female pairs). These traits were assayed as broad tests for adaptation to the different treatment conditions and to determine whether adaptation occurred at the expense of reduced performance in the other environments (i.e. a cost to adaptation). The second category of traits pertained to water balance, specifically tolerance of prolonged desiccation stress, rate of water loss, and cuticular hydrocarbon (CHC) profiles. These traits were examined because altered aPO_2_ environments may affect water balance by modifying respiratory water loss via the degree of spiracle opening [13] or by modifying the cuticular water loss by altering the relative abundances of longer chain-length CHCs [16], [17]. Body mass was also measured because divergence in it may affect the interpretation of changes in these other traits. The final category of traits was associated with metabolism and activity, in particular whole-animal routine metabolic rate (RMR), locomotor activity, and mitochondrial citrate synthase (CS) activity. These traits were measured because of their potential association with increased performance of the hypoxic-evolved populations.

## Results

Due to the extinction of the original four hyperoxic populations after three generations when maintained at 60% O_2_, this treatment was restarted, at 40% O_2_, five generations after the hypoxic and normoxic treatments using flies from the same stock (see Methods). This difference is denoted parenthetically where relevant; for example, 6(1) indicates generation six of the hypoxic and normoxic populations and generation one of the hyperoxic populations.

### Performance/Fitness

**Figure 1:**
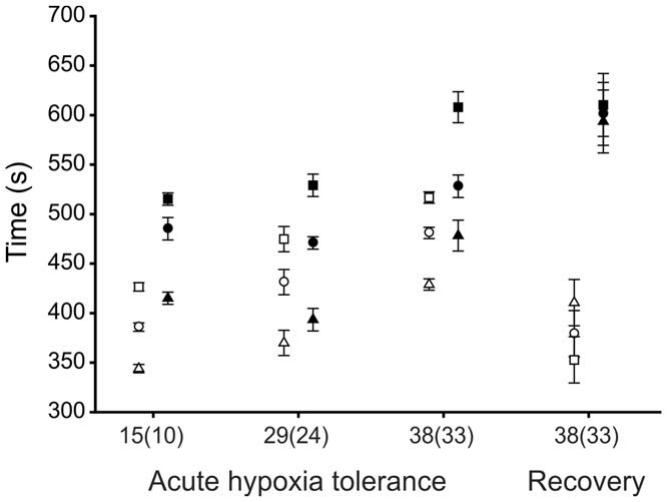
Variation among treatments in the tolerance of, and recovery from, acute hypoxia at various generations. Male (open symbols) and female (closed symbols) *D. melanogaster* were evolved under normoxic (circles), hypoxic (squares) or hyperoxic (triangles) conditions and tolerance was assayed after 15(10), 29(24) and 38(33) generations of experimental evolution, and recovery after 38(33) generations. Hyperoxia-exposed flies (generation numbers in parentheses) were started five generations after the other treatments. All flies were raised in a common normoxic environment for two generations prior to conducting the assay. Treatment means ± SEM are displayed. Treatment effects on acute hypoxia tolerance are significant at every time point and the three treatments differ significantly from one another in post-hoc comparisons (p<0.001 in all cases). Sexes do not differ at any time point (p>0.15 in all cases). Recovery time differed between sexes (p = 0.039) but not between treatments (p = 0.849), although the sex×treatment interaction approached significance (p = 0.075).

Tolerance of acute hypoxia, measured as the time to incapacitation, differed significantly among treatments but not between the sexes in three separate assays conducted at generation 15(10) (treatment: F_2,6_ = 22.73, p<0.001; sex: F_1,3_ = 3.14, p = 0.175), generation 29(24) (treatment: F_2,6_ = 145.29, p<0.001; sex: F_1,3_ = 3.52, p = 0.157) and generation 38(33) (treatment: F_2,6_ = 24.93, p = 0.001; sex: F_1,3_ = 0.70, p = 0.150). Within sexes, post-hoc analyses revealed that all three treatments differed significantly from one another within each generation, with hypoxia-evolved flies being more tolerant than the normoxia-evolved flies which were more tolerant than the hyperoxia-evolved flies (Fig. 1). Recovery time after succumbing to severe hypoxia (with continued exposure to anaerobic gas for a total of 15 min.), measured at generation 38(33) as the time taken to return to a standing position, differed significantly between the sexes (F_1,3_ = 12.26, p = 0.039) with males recovering faster than females (Fig. 1). Recovery times did not differ significantly between treatments (F_2,6_ = 0.17, p = 0.849), although the pattern in males mirrored the tolerance results (i.e. recovery times of hypoxic-evolved males<normoxic-evolved males<hyperoxic-evolved males; Fig. 1). Females, in contrast, differed little in recovery times among treatments, generating a sex×treatment interaction that approached significance (F_2,6_ = 4.10, p = 0.075).

In contrast to the increased performance of hypoxia-evolved flies under severe hypoxia, there was no evidence of any performance differences among treatments when experiencing increased oxidative stress (Fig. S1). When assayed as longevity under 100% O_2_ at generation 15(10), mortality was best described by a Gompertz model in males and a Gompertz-Makeham model in females (see Methods) in which α is the baseline mortality rate, *β* is the rate of senescence, and *c* is the fixed rate of age-independent mortality (Gompertz-Makeham model only) [18]. None of these underlying parameters differed significantly among treatments in either sex (Table 1).

**Table 1:** Treatment effects on Gompertz (male) and Gompertz-Makeham (female) mortality parameters when measured under 100% O_2_ at generation 15(10 hyperoxia).

	α		*β*		*c*	
Sex	F ratio	p value	F ratio	p value	F ratio	p value
Male	1.31	0.317	0.86	0.454		
Female	1.73	0.230	3.59	0.071	2.56	0.131

Note: F ratios from nested ANOVA, separately by sex, testing for treatment differences (*d.f.* = 2,9 in all cases). α is the baseline mortality rate, *β* is the rate of senescence, and *c* is the fixed rate of age-independent mortality (estimated in females only).

**Figure 2:**
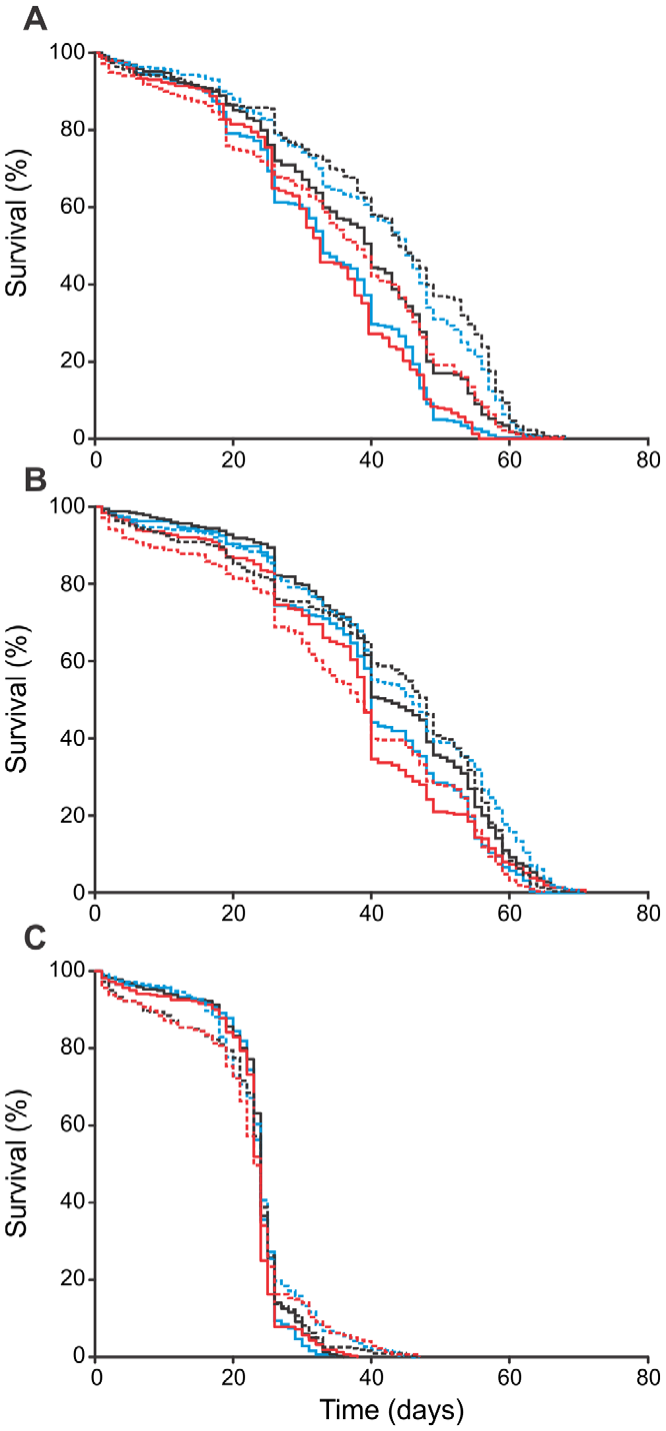
Mortality as measured in a reciprocal transplant assay among treatment environments at generation 35(30 hyperoxia). Mortality in male (solid) and female (dashed) *D. melanogaster* from populations evolved under normoxic (black), hypoxic (blue) and hyperoxic (red) treatment environments and then assayed under A) hypoxic, B) normoxic, and C) hyperoxic conditions. All populations were raised in a common normoxic environment for two generations prior to conducting the assay. Treatment means of the four replicate populations are shown for clarity. The underlying logistic mortality parameters (*α*, *β*, *s*) did not differ between treatments, nor were there any significant interactions involving treatment. Significant differences were detected as a function of sex, assay environment, and their interaction (Table 2). Longevity was measured for 320 individuals/sex/treatment (80 individuals/sex/population) within each assay environment (see Methods).

As direct tests for adaptation, longevity and productivity were measured separately in full reciprocal transplants among the three treatment environments (i.e. hypoxia, normoxia, and hyperoxia). With respect to longevity, assayed in generation 35(30), the resulting mortality curves for males and females were both best described by a logistic model in which *α* is the baseline (i.e. initial) mortality, *β* is rate of senescence (i.e. the rate at which mortality increases with age), and *s* is the rate at which mortality decelerates in late life [18]. None of these mortality parameters differed significantly among the evolved treatments nor were there any significant interactions involving treatment (Table 2; Fig. 2). Environmental effects were detected with all three longevity parameters differing between the assay environments, and the sexes also differed in senescence rate (i.e. *β*). For the parameters of baseline mortality and senescence, the sex×environment interaction was also significant, revealing parameter-specific effects that were sex and environment specific (Table 2).

**Table 2:** Variation in logistic mortality parameters between sexes, treatments, and environments in a reciprocal transplant longevity assay at generation 35(30 hypoxia).

		*α*			*β*			*s*		
Source of Variation	*d.f.*	F ratio	p value	Post-hoc	F ratio	p value	Post-hoc	F ratio	p value	Post-hoc
Sex	1,9	2.92	0.122		14.26	**0.004**	m>f	0.23	0.642	
Treatment	2,9	1.33	0.311		0.09	0.912		0.09	0.916	
Environment	2,18	4.89	**0.020**	5>40;21 = 40,5	40.42	**<0.001**	40>21 = 5	23.75	**0.019**	40>21 = 5
Treat×Envt.	4,18	0.52	0.723		0.16	0.999		0.83	0.523	
Sex×Treat.	2,9	2.72	0.119		1.18	0.351		2.48	0.139	
Sex×Envt.	2,18	4.47	**0.027**	5m = 21f = 5f> 40m; 40f = 21m = 40m;5m = 21f = 5f = 40f = 21m	7.74	**0.004**	40m>40f>21m = 21f = 5f = 5f	0.79	0.470	
Sex×Treat.×Envt.	4,18	0.51	0.731		0.72	0.590		1.22	0.337	

Note: F ratios are from separate nested ANOVA of the parameters from a logistic mortality model. Treatment is the environment in which the populations were evolved and environment indicates the conditions under which longevity was assayed. *α* is the initial mortality rate, *β* estimates the rate of senescence, and *s* is the rate of mortality deceleration. Results of post-hoc tests of significant effects (bold) are given (m = males, f = females, 5 = hypoxia, 21 = normoxia, 40 = hyperoxia).

**Figure 3:**
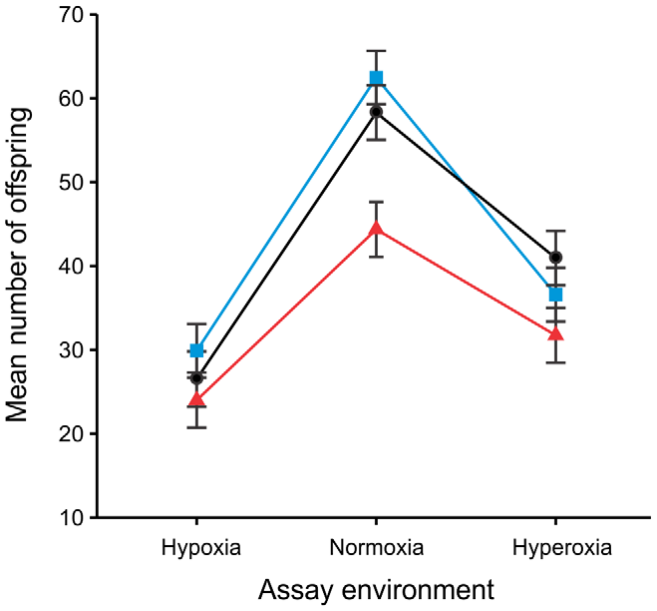
Productivity as measured in a reciprocal transplant among treatment environments at generations 35–36(30–31 hyperoxia). Productivity, measured as the number of adult offspring produced by a male-female pair after 14 days, in *D. melanogaster* populations evolved under normoxic (black circles), hypoxic (blue squares) and hyperoxic (red triangles) treatment environments and then assayed under hypoxic, normoxic, and hyperoxic environmental conditions. All populations were raised in a common normoxic environment for two generations prior to conducting the assay. The main effects of assay environment (p<0.001) and treatment (p = 0.022) and were significant, but not their interaction (p = 0.186). Post-hoc analyses reveal significant differences among all three assay environments, and a significantly reduced productivity in the hyperoxia treatment relative to the other two.

Productivity, assayed in two blocks in generations 35(30) and 36(31), differed significantly between the evolved treatments (F_2,9_ = 6.02, p = 0.022), with a post-hoc analysis revealing that the hyperoxia-evolved populations had a significantly lower productivity overall compared to the other two treatments (Fig. 3). Assay environment also had a significant effect (F_2,18_ = 58.76, p<0.001), with hypoxia and hyperoxia reducing productivity by 51% and 34%, respectively, compared to normoxia. All three assay environments were significantly different in a post-hoc test. The treatment×environment interaction was non-significant, providing no evidence that the evolved responses varied among assay environments (F_4,18_ = 1.73, p = 0.186).

### Water balance and CHC profiles

**Figure 4:**
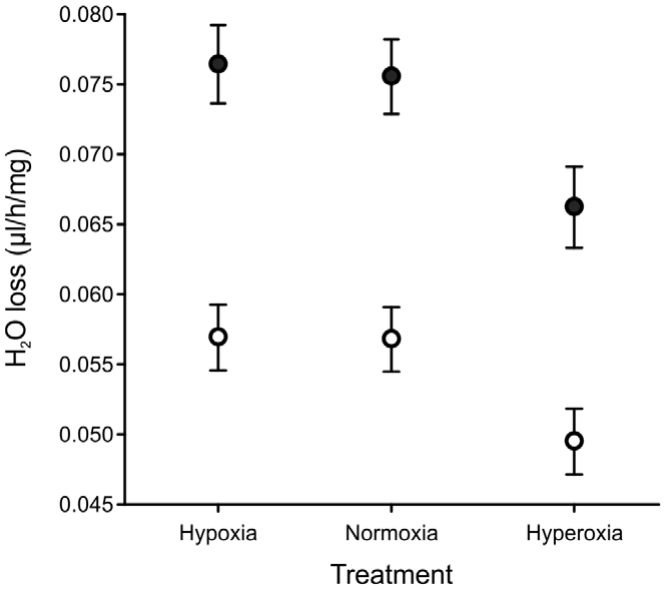
Variation among treatments in water loss rates under normoxic conditions as measured at generation 29(24 hyperoxia). Male (solid) and female (open) *D. melanogaster* evolved under normoxic, hypoxic, or hyperoxic conditions and were then raised in a common normoxic environment for two generations prior to conducting the assay. Treatment means ± SEM of replicate populations are shown for clarity. Body-mass corrected water loss rates were lower in females than in males (p = 0.0002), but there was no significant treatment effect (p = 0.099) nor sex×treatment interaction (p = 0.931).

Survival under prolonged desiccation stress at generation 29(24) was best described by a logistic model in males and a Gompertz model in females in which *α* is the baseline mortality rate, *β* is the rate of senescence, and *s* is the rate of mortality deceleration (logistic model only) [18]. None of these mortality parameters differed significantly among treatments in either sex (Table 3; Fig. S2). When mass-specific rates of water loss were measured directly under normoxic conditions at generation 29(24), males and females differed significantly (F_1,9_ = 36.77, p<0.001), with males exhibiting a higher water loss rate than females (Fig. 4). Hyperoxic-evolved flies also tended to have lower values overall although this was not sufficient to generate a significant treatment effect (Fig. 4, F_2,9_ = 3.01, p = 0.099), and there was no evidence of a sex×treatment interaction (F_2,9_ = 0.07, p = 0.931). At generation 15(10), there were no evolved differences between treatments in body size (F_2,9_ = 2.08, p = 0.181), suggesting that this trend of lower mass-specific water loss rate of hyperoxic-evolved flies cannot be accounted for by an effect of body mass. Females were heavier than males (F_2,9_ = 1283.74, p<0.001), however, and there was no evidence of a sex×treatment interaction (F_2,9_ = 2.00, p = 0.196).

**Table 3:** Treatment effects on logistic (male) and Gompertz (female) mortality parameters when measured under severe desiccation stress at generation 15(10 hyperoxia).

	α		β		*s*	
Sex	F ratio	p value	F ratio	p value	F ratio	p value
Male	1.71	0.235	0.67	0.534	0.73	0.508
Female	1.30	0.319	0.43	0.662		

Note: Results of nested ANOVA, separately by sex, testing for treatment differences (*d.f.* = 2,9 in all cases). *α* is the baseline mortality rate, *β* is the rate of senescence, and *s* is the rate of mortality deceleration (estimated in males only).

With respect to cuticular hydrocarbons (CHCs), 38 were consistently detected in females and 28 in males (Fig. S3). In females, the relative concentration of only seven of these 38 differed significantly between treatments and none of these remained significant after correction for multiple comparisons (Table S1). In contrast, in males 11 of 28 CHCs differed significantly between treatments in relative concentration and ten of these remained significant after correction for multiple comparisons. These differences were scattered fairly evenly across carbon chain-lengths, as indicated by their retention times, and the treatments effects showed no consistent directionality (i.e. hypoxia>hyperoxia or vice versa; Table S1).

### Metabolism and locomotor activity

**Figure 5:**
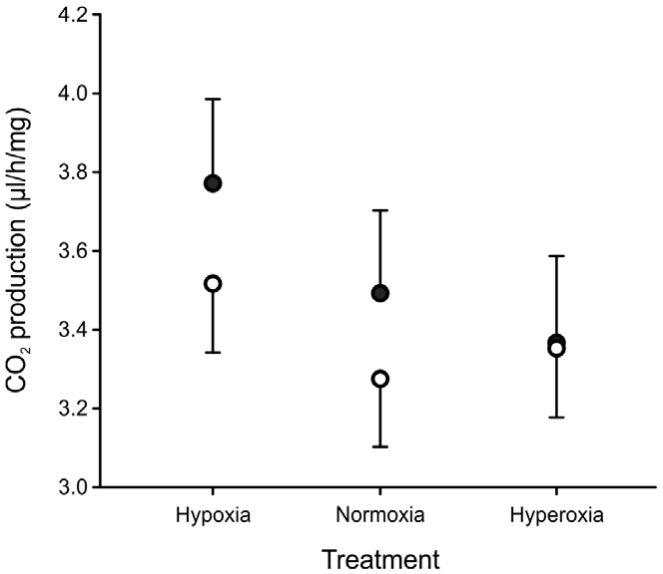
Variation among treatments in routine metabolic rate, measured as CO_2_ production under normoxic conditions at generation 29(24 hyperoxia). Male (filled) and female (open) *D. melanogaster* were evolved under normoxic, hypoxic or hyperoxic conditions and were then raised in a common normoxic environment for two generations prior to conducting the assay. Treatment means ± SEM of replicate populations are shown and, for clarity, error bars are omitted in one direction. There were no significant differences between the sexes (p = 0.417) or treatments (p = 0.163), and no evidence of a sex×treatment interaction (p = 0.862).

**Figure 6:**
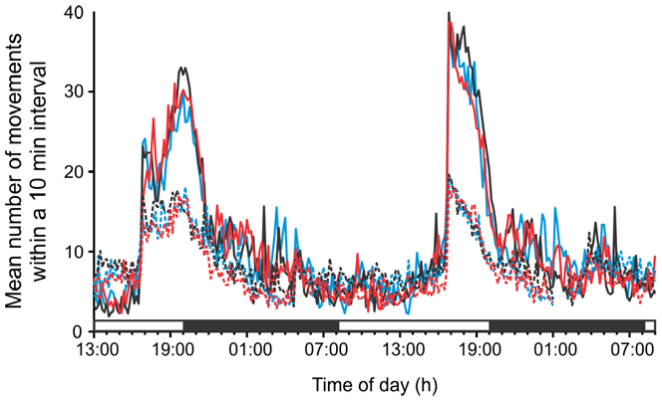
Variation among treatments in male and female activity levels as measured under normoxia at generation 49(44 hyperoxia). Mean number of movements within a 10 min period for male (solid) and female (dotted) *D. melanogaster* populations evolved under normoxic (black), hypoxic (blue), or hyperoxic (red) conditions and then raised in a common normoxic environment for two generations prior to conducting the assay. Treatment means are shown for clarity. The upper bar indicates the times at which the lights are on (white) and off (black) in the incubator. Activity varied significantly through time in a sex-specific manner, although treatments did not differ significantly (Table 4).

**Figure 7:**
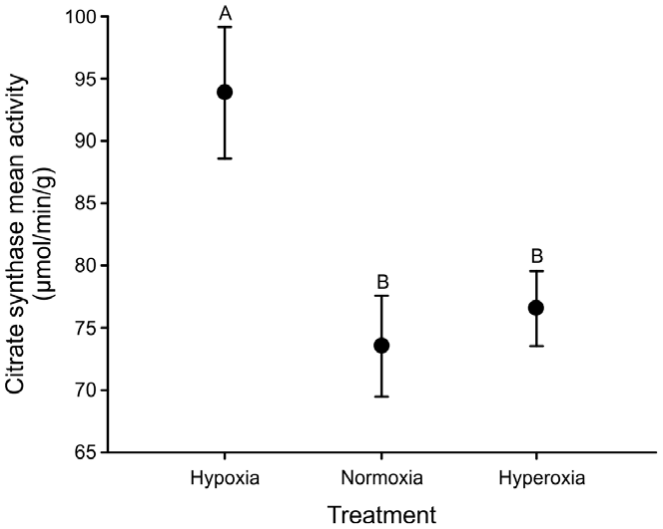
Variation among treatments in mitochondrial citrate synthase activity as measured in males at generation 29(24 hyperoxia). *D. melanogaster* populations evolved under normoxic, hypoxic, or hyperoxic conditions and were then raised in a common normoxic environment for two generations prior to conducting the assay. Treatment means ± SEM of replicate populations are shown for clarity. The treatment effect was significant overall (p = 0.002) and those not sharing a letter are statistically different in a post-hoc comparison.

When assayed under normoxic conditions at generation 29(24), there were no significant differences in mass-specific routine metabolic rate between the treatments (F_2,6_ = 2.23, p = 0.163) or sexes (F_2,6_ = 0.72, p = 0.417), and no evidence of a sex×treatment interaction (F_2,9_ = 0.15, p = 0.862; Fig. 5). Although activity was not monitored during this assay, a separate assay of individual flies at generation 49(44) revealed no difference among treatments in total daily locomotor activity (sum of all movements over a 24 h period) under normoxic conditions in females (F_2,9_ = 0.28, p = 0.76; total number of movements ± SE, hypoxic treatment: 2418±417, normoxic treatment: 2237±141, hyperoxic treatment 1901±368) nor in males (F_2,9_<0.01, p = 1.00; total number of movements ± SE, hypoxic treatment: 2430±502, normoxic treatment: 2435±388, hyperoxic treatment 2430±506). A more detailed repeated measures comparison of the circadian activity profiles across a 42 h period also revealed no significant treatment effects, nor were any of the interactions involving treatment significant (Table 4; Fig. 6). Activity did cycle diurnally, with levels rising sharply in both sexes around 16:50 and then declining more gradually from around 21:00–22:00. Male activity was much higher than female activity during these peak times, generating a strong sex×time interaction (Table 4). Finally, mitochondrial citrate synthase activity differed significantly between treatments (F_2,9_ = 13.49, p = 0.002), with a post-hoc test revealing that populations from the hypoxia-evolved treatment had significantly higher activity levels compared to those from the other treatments (Fig. 7).

**Table 4:** Variation in male and female activity levels as measured under normoxic conditions at generation 49(44 hyperoxia).

Source of Variation	*d.f.*	F ratio	P value
Treatment	2, 9	0.07	0.934
Sex	1, 9	1.84	0.208
Sex×Treatment	2, 9	0.16	0.853
Time	249, 2241	47.08	<0.001
Time×Treatment	498, 2241	0.57	0.797
Sex×Time	249, 2241	12.90	<0.001
Sex×Time×Treatment	498, 2241	0.79	0.771

Note: Results of a full-factorial repeated measures ANOVA of male and female locomotor activity data (Fig. 6). Tests of within-subject factors (sex and time) employ a Greenhouse-Geisser epsilon-adjustment [43].

## Discussion

The goal of the present study was to characterize the evolutionary response to altered aPO_2_ among replicate populations of *D. melanogaster*, including direct tests for adaptation to both hypoxic and hyperoxic environments and the subsequent assessment of the traits underlying adaptation. Ultimately, our intent was to provide insight into the potential physiological mechanisms responsible for increased performance in these environments. The hypoxic and hyperoxic environments we employed had substantial negative impacts on productivity, reducing it by 51 and 34% respectively (relative to that in the normoxic environment) in the control populations when raised for a single generation in these environments (Fig. 3). Cleary, these environments were stressful, suggesting that at least initially the populations were not well adapted to them.

### aPO_2_ modification of acute hypoxia tolerance and recovery

Tolerance of acute hypoxia evolved in response to altered aPO_2_ in our experiment. Changes in tracheal morphology could underlie these differences in performance given their potential role as an internal O_2_ store [19] and their function in O_2_ delivery to tissues during acute hypoxia. Environmentally-induced changes in larval tracheal morphology have previously been observed in response to altered aPO_2_, including an increase in tracheal diameter and the number of fine terminal branches [8], [20]. Consistent with changes in tracheal morphology increasing O_2_ storage capacity in our populations, there was a tendency towards faster recovery from acute hypoxia in our hypoxia-evolved males. However, changes in tracheal morphology in response to altered aPO_2_ did not appear to evolve in a recent independent selection experiment, as inferred from measurements of critical PO_2_ and maximal tracheal conductance [15]. This suggests that the link between changes in tracheal morphology and whole-animal performance is more complex than simply altered O_2_ storage. In our experiment, the correlated evolution between acute hypoxia tolerance and recovery in males, but not in females, also suggests that O_2_ storage is not the common link.

### Longevity and aPO_2_

Our results support the plastic responses (both developmental and acclimatory) of single-generation exposure to differing PO_2_, as previously reported [14]. Exposure to elevated aPO_2_ decreased lifespan to a similar extent across in all populations, irrespective of evolved treatment, with 100% O_2_ greatly reducing adult lifespan as compared to 40% O_2_. However, unlike a previous report [14], we did not observe any increase in lifespan associated with 10% O_2_ exposure. Our results also provide no evidence of evolved differences in longevity under the different selection treatments. This is perhaps not surprisingly given that the selection regimes imposed strict 14 day lifespans, making longevity beyond that age essentially a neutral trait.

### Adaptation of hypoxia-evolved populations

Tolerance of acute hypoxia was highest in the hypoxia-evolved populations (Fig. 1) and this difference was an evolved response (populations were raised for two generations in a common normoxic environment prior to all assays). Males from these populations also tended to recover faster from hypoxic incapacitation, although females showed no such effect, thereby generating a borderline non-significant sex×treatment interaction (p = 0.075). The parallel response of these populations indicates adaptation to the hypoxic environment because genetic drift is unlikely to cause such a consistent response in correlation with environment. Improved tolerance of severe hypoxia has presumably arisen as a consequence of selection for increased performance in the hypoxic (i.e. 5–10% O_2_) environment under which these populations evolved. Surprisingly, the adaptive benefit of this increased performance in these hypoxia-evolved population was not detectable in the fitness components measured (i.e. productivity and longevity). There are at least two potential explanations for this apparent discrepancy. First, these assays may not have captured the relevant fitness components underlying increased performance in the hypoxic environment. Indeed, lifetime fitness is difficult to measure empirically in laboratory populations [21], [22] and our assays did not include all components contributing to it. For example, reproductive success was not measured yet is often considered to be the major component of male fitness in organisms such as *D. melanogaster* in which males contribute only genes to their offspring. Therefore, it is possible that increased performance under hypoxic conditions resulted from an increase in male pre- and/or postcopulatory sexual fitness. Similarly, although productivity captures a substantial component of lifetime fitness, including female fecundity and the survival to emergence of both sexes, female mate choice (cryptic or overt) may also be important (e.g. [23]) yet was prevented in this assay. The second potential explanation is that the fitness measures were inappropriate because they were measured under conditions that did not exactly replicate those experienced during experimental evolution, for example involving differences in larval density and hence competitive environments. It is possible, therefore, that the hypoxia-evolved population would outperform the others under conditions more representative of those they experienced during experimental evolution. Further work would be needed to distinguish these possibilities.

### Adaptation of hyperoxia-evolved populations

In contrast to the results for the hypoxia-evolved populations, there is no direct evidence of increased performance of the hyperoxia-evolved populations under hyperoxic conditions. Specifically, hyperoxia-evolved flies showed no reduction in mortality under prolonged and severe oxidative stress (i.e. 100% O_2_) and no increase in productivity or longevity (relative to the controls) under hyperoxia (40% O_2_). Nonetheless, a consistent reduction in hypoxia tolerance of all four replicate hyperoxia-evolved populations was observed (Fig. 1). Such decreased performance could have arisen in two ways. First, it may have resulted from a general reduction in fitness caused by inbreeding depression [24]. Indeed, productivity was also lowest overall in the hyperoxia-evolved populations, independent of rearing environment, consistent with this hypothesis. However, decreased performance of the hyperoxia-evolved populations was not detected in other performance measures conducted in hyperoxic and xeric environments. In addition, census population sizes remained large (*N*>1000) and similar to the other treatments throughout the experiment (M. Charette, personal observation). A more likely explanation is therefore that decreased hypoxia tolerance is an evolutionary cost of adaptation to hyperoxic conditions, and as discussed above for hypoxia, the design of the hyperoxic performance/fitness assays may have been inappropriate to detect this adaptation.

### Effects of aPO_2_ on desiccation and water loss

In the hyperoxia-evolved populations, both males and females tended to have a decreased rate of water loss (Fig. 4), although the difference was not significant (p = 0.099). Our measure of water loss combined both cuticular and respiratory routes of exit, meaning that changes in either (or both) could underlie this trend. Although CHCs also evolved in our experiment, only males differed significantly among treatments and we observed no consistent increase in long chain-length CHCs in the hyperoxia-evolved populations, as would be expected if the reduced rate of water loss in these populations was due to CHC-mediated changes in cuticular water loss (e.g., [16], [17]). In addition, the desiccation tolerance assay, in which adults were exposed to 0% RH until they succumbed, did not reveal any observable changes in desiccation tolerance, although this environment may have been so severe as to make it difficult to detect the effects of relatively small differences in rates of water loss.

Water loss also occurs via respiration and is directly affected by the size and frequency of spiracle opening [13]. Spiracle opening is reduced during exposure to hyperoxia, a behaviour that is termed the *hyperoxic-switch* and which is thought to limit oxidative damage and consequently cause a reduction in respiratory water loss [13]. That the hyperoxia-evolved populations tended to have a decreased rate of water loss is consistent with this behaviour being adaptive in perpetually hyperoxic conditions.

### Evolutionary consequences of hypoxia on metabolism

Increased hypoxia tolerance in our hypoxic-evolved populations was accompanied by an increase in CS activity, as has also been observed in other species inhabiting hypoxic conditions [25], [26], [27]. This evolved response supports the hypothesis that populations inhabiting perpetually hypoxic environments overcome challenges related to hypoxia by increasing their aerobic capacity [28]. The exact mechanism(s) by which increased CS activity (and the inferred increase in mitochondrial volume density) increases performance in hypoxia is not clear [2]. Moreover, recent work has linked the ability to recover from hypoxia to the capacity of mitochondria to oxidize anaerobic end-products [3]. Our observed trend toward faster recovery of hypoxic-adapted males, in combination with their increased mitochondrial CS activity, is consistent with this connection.

Higher rates of CS activity did not correlate with higher RMR in hypoxia-evolved flies (Fig. 5), a result which is consistent with previous measures of RMR in hypoxia/hyperoxia-evolved *D. melanogaster* [15]. In addition, metabolic rate and adult locomotor activity did not differ among hypoxia and hyperoxia evolved populations (Fig. 5, 6). Thus, the increased CS activity found in hypoxia-evolved flies did not impact routine metabolic rate of adult flies, indicating that an increased mitochondrial capacity was likely not associated with an overall change in energy turnover rate [29].

### Conclusions

Multigenerational exposure to hypoxia resulted in the evolution of increased animal performance during exposure to hypoxic conditions. Conversely, the hyperoxia-evolved populations exhibited an evolved cost in terms of performance under hypoxic conditions. Hypoxia-evolved populations also exhibited an evolved increase in CS activity, which might in part explain their increased performance in low aPO_2_ environments. While there was no detectable increase in the productivity or longevity of evolved treatments when assayed in their respective experimental environment, it is possible that our assays failed to capture the relevant fitness component. This study has provided several examples of evolved traits underlying improved performance in hypoxic conditions. These traits provide a basis for future studies examining the effects of aPO_2_ on physiological tolerances and performance.

## Materials and Methods

### Derivation and maintenance of experimental populations

Twelve replicate experimental populations were independently derived from a previously described outbred and laboratory-adapted stock population [30] via the ‘random’ selection of approximately 400 individuals. These populations were maintained separately, with no gene-flow among them, via non-overlapping 14-day generations in groups of ten vials per population (each vial containing 10 mL of standard cornmeal-based food with live yeast sprinkled on top). Every generation, adult offspring from the ten vials were mixed without anaesthesia via transfer to a common bottle. Approximately 30 randomly chosen adults, unidentified by sex, were then aspirated into in each of ten new vials and allowed to lay eggs for 48 h before being discarded.

Four populations were assigned to each of three treatment environments of differing atmospheric composition: hypoxia (5% O_2_:95% Ar), normoxia (ambient air containing approximately 21% O_2_, as supplied by the building's compressed air system), and hyperoxia (40% O_2_:60% Ar). Argon was used instead of nitrogen in the hypoxia and hyperoxia treatments because the latter has been found to increase chill coma recovery time [31], suggesting that it may have direct effects on performance. Individuals within each population experienced their respective treatment environments throughout their entire lifecycle. The normoxic environment had the same ambient atmospheric composition as that experienced by the stock from which the experimental populations were derived and therefore acts as a control for the maintenance protocol. The hypoxia and hyperoxia environments differed from it only in their atmospheric composition such that differences among them indicate responses to the atmospheric treatments. As a result of the extinction of all four of the hyperoxic populations (originally maintained at 60% O_2_: 40% Ar), this treatment was restarted (at 40% O_2_: 60% Ar) five generations after the hypoxic and normoxic treatments using flies from the same stock.

Atmospheric compositions were manipulated by housing the populations in 19 cm×19 cm×13 cm acrylic-plastic chambers with separate inlet and outlet nozzles and a removable airtight lid. One chamber was used for each of the three atmospheric treatments and all four populations within a treatment were housed together within a particular chamber (i.e. four populations of ten vials per population). The inlet of each chamber was connected via plastic tubing to a mixing system that delivered the appropriate atmospheric composition. Oxygen/argon mixes for the hypoxic and hyperoxic treatments were achieved using flowmeters (Gilmont Instruments Inc, Barrington, USA), with the appropriate mixture determined by measuring the O_2_ partial pressure (PO_2_) inside a vial within a chamber using a FOXY AL 300 coated fiber optic O_2_ sensor (Ocean Optics, Dunedin, USA). Gas mixtures and compressed air were delivered to the chambers at a flow of 200 mL/min to remove excess moisture, yielding relative humidities within each chamber of approximately 60% (range 50–70%). Humidity was subsequently monitored throughout the experiment via a digital hygrometer (model 63–1013, Tandy Corporation, Fort Worth, TX) placed directly within each chamber. All three chambers were housed within a single Caron 6030 (Caron, Marietta, USA) constant-temperature incubator at 25°C with a 12L∶12D light cycle.

PO_2_ was monitored in all treatments for the first five generations and then discontinued. A later measurements at generation 20/15 revealed that it had increased to 10% O_2_ in the hypoxic treatment. Subsequent measurements at generations 25/20, 30/25, and 35/30 confirmed that this was stable. When and how this value increased over these generations is not known and for simplicity throughout we refer to the hypoxic treatment as 10% O_2_, although in reality the environment varied between 5–10%.

### ‘Common gardens’ and reciprocal transplants

To isolate and characterize the evolutionary response to these treatment environments, a variety of traits were assayed on individuals from these populations at various times during experimental evolution, as described below. In all cases, prior to assaying a particular trait a sample of individuals from all populations were first raised for two generations in a common normoxic environment, thereby removing any environmental differences including cross-generational maternal effects. Conditions during these two generations matched those used in the maintenance of the normoxic treatment (i.e. 30 adults per vial on the same food, held at 25°C, 50% relative humidity, 12L∶12D photoperiod), except that the chambers housing the vials were open to the ambient air instead of having ambient air flowing through them at 200 mL/min (from the building compressed air system).

Unless otherwise indicated, individuals for use in assays were then collected 24 h after clearing the vials (i.e. as one day-old potentially mated adults) using light CO_2_ anaesthesia and stored in groups of ten same sex individuals in vials containing 5 mL of cornmeal media until their use in an assay. The majority of the traits were then assayed under common normoxic conditions (i.e. ‘common garden’ assays) separately by sex. Adaptation in such assays is indicated by consistent trait differences among treatments, treating populations as replicates (i.e. parallel evolution), because genetic drift is unlikely to produce concerted change, correlated with environment, in multiple, independent populations [32], [33]. For two traits (i.e. productivity and longevity), we employed a full reciprocal transplant in which all populations were assayed in all three treatment environments, providing additional insight into environmental effects and potential costs of adaptation. Statistical analyses were specific to each assay due to their different designs (described below) but in all cases accounted for the fact that populations are the independent unit of replication in tests for treatment effects and individuals (or groups thereof) within populations represent subsamples. Tukey's post-hoc tests were employed in the presence of a significant overall effect to explore the differences between levels of the effect. JMP 7 (SAS Institute, Cary, NC) was used for all statistical analyses with the exception of the locomotor activity assay, which was analyzed using SAS version 9.2 (SAS Institute, Cary, NC).

### Performance/Fitness traits

#### Acute hypoxia tolerance and anoxic recovery

At generations 15(10), 29(24) and 38(33), tolerance of acute hypoxia was assayed using the protocol described in Dawson-Scully *et al.* [34]. In brief, groups of ten 2–3 day-old same sex individuals were placed together in a vial capped with a porous foam plug. Six vials were placed together within a 600 mL beaker and covered with Parafilm®, with two vials originating from a single population from each of the three treatments. This generated four blocks, each representing a unique combination of one population from each treatment (e.g., denoting the treatments L, N, and H for low, normal and high O_2_, each having four replicate populations, then block 1 included populations L1, N1, and H1, block 2 included populations L2, N2, and H2, and so on). Three replicate beakers were performed per block and sex, all using new groups of flies. During each replicate, the beaker was flooded with Ar at 600 mL/min via a foam-tipped needle inserted through the Parafilm®. As with the evolution experiment itself, Ar was used because nitrogen has been found to increase chill coma recovery time [31]. Incapacitation times (in seconds) of individuals were recorded visually with the aid of the computer program MAMER (Multi-Arena-Multi-Event-Recorder, developed by C.A.L. Riedl and freely available at http://cfly.utm.utoronto.ca/MAMER). Timing was started when the Ar was introduced into the system and incapacitation was indicated by a number of small convulsions and a loss of adhesion of the fly to the surface. In addition, recovery times following incapacitation were measured using different individuals at generation 38(33) by leaving the flies in the chamber while Ar gas was continuously injected for 15 min. The Parafilm® was then removed and the vials were placed on the counter, allowing them to equilibrate with the ambient air. Individual recovery times were recorded visually as the time (in seconds) at which a fly righted itself on all six legs.

Block means for each sex and treatment were created by averaging all the population subsamples (i.e. replicate vials and then beakers). The statistical analysis was conducted separately by generation and employed a non-additive mixed linear model for a factorial randomized complete block design:
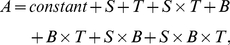
(1)in which *A* is the average incapacitation or recovery time of a group of individuals of a particular sex (*S*) from treatment (*T*) within block (*B*). Block is a random effect and represents the blocking of the experimental units (populations) into four unique sets, each consisting of one population each from the hypoxic, normoxic, and hyperoxic treatments respectively. Sex and treatment were fixed effects and their F ratios were constructed using the mean square of their respective interaction with block as the denominator, and the sex×treatment interaction was tested over the mean square of the three-way interaction [35]. As with all unreplicated versions of such a design, there is no test of the block effect or of any of the interaction terms involving it [35].

#### Tolerance of prolonged oxidative stress

To test for differences in the capacity to prevent and/or withstand oxidative stress, six groups of ten day-old same-sex individuals from generation 15(10) were stored in vials with 5 mL standard food and then placed in a chamber at 100% O_2_. Following previous studies [36], 100% O_2_ was chosen to induce maximal oxidative stress, thereby making treatment effects easier to detect. Individual survival was recorded every 12 h by visual inspection, with each vial scored for deaths until all flies had died. Transfer to fresh media was not necessary because all flies died in under a week (Fig. S1). From these individual time-to-death data, a series of nested likelihood ratio tests were used to determine the best fit mortality model from the Gompertz-family for each population, fit separately by sex using the maximum likelihood method implemented in the program WinModest 1.0.2 [18]. The best-fit model shared by the majority of populations was then used in subsequent analyses.

For females, in the majority of populations mortality at age *x* (*μ_x_*) was best described by the Gompertz-Makeham model, *μ_x_* = *α*e*^βx^*+*c*, in which *α* is the initial mortality rate, *β* is the rate of increase in mortality, and *c* is the fixed rate of age-independent mortality. In males, the simpler Gompertz model, *μ_x_* = *α*e*^βx^*, tended to fit best. We therefore used WinModest to estimate these parameters separately by sex for each population. Differences in the parameter values (*α*, *β* and *c* in females; *α* and *β* in males) between treatments were then tested using a mixed model in which population was a random effect nested within the fixed effect of treatment. The F ratio for the treatment effect was constructed using the mean square of population nested within treatment as the denominator [35]. False discovery rate (FDR) correction was used, with α = 0.05, to account for multiple comparisons [37].

#### Longevity

At generation 35(30), a full reciprocal transplant assay was conducted in which the time-to-death for adults from all 12 populations were assayed under conditions of each of the three treatment environments (i.e. hypoxia, normoxia and hyperoxia). For each population, eight replicate vials of 10 same-sex one-day old adults were placed in each of three chambers that received one of the three environmental gas mixtures matching those used during experimental evolution. Vials were scored for death once daily until all flies had died. Flies were transferred to new vials with fresh food weekly. The resulting individual time-to-death data were analyzed with WinModest 1.0.2, as previously described. Mortality was best described in the majority of populations of each sex by a logistic model, *μ_x_* = *α*e*^βx^*/(1+(*αs*+*β*)(*e^βx^*−1)), where *α* and *β* are as previously described and *s* is the rate of mortality deceleration. Parameters of this model (*α*, *β* and *s*) were therefore estimated separately for each sex and population and analyzed via a partly nested split plot design:
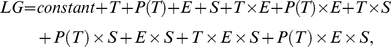
(2)in which *LG* is the parameter (*α*, *β* or *s*) describing the mortality curve of individuals from population (*P*) nested within treatment (*T*) within assay environment (*E*) of a given sex (*S*). Treatment, environment and sex were fixed effects and population was a random effect. The F ratio for the treatment effect was constructed using the mean square of population nested within treatment as the denominator. The F ratios for the environment and environment×treatment effects employed the mean square of environment×population(treatment), while sex and sex×treatment employed the mean square of sex×population(treatment) as the denominator. The F ratios for the three way interaction and the sex×environment term used sex×environment×population(treatment) as the denominator [35].

#### Productivity

At generation 35(30) and 36(31), productivity was measured in a reciprocal transplant assay by counting the number of adult offspring produced by replicate male-female pairs when raised in each of the three treatment environments. Productivity therefore represents a combined measure of the fecundity of a particular female and the egg-to-adult survivorship of her male and female offspring. The assay was conducted in two blocks, each consisting of 50 replicate pairs from each population raised in each environment. Gases were mixed as described previously in the maintenance protocol for the evolution experiment. Day-old male-female pairs were collected from each experimental population via light CO_2_ anaesthesia and were immediately placed together in a vial containing 10 mL of standard cornmeal media for two days, after which they were discarded. Mirroring the maintenance of the populations during experimental evolution, fourteen days after setting-up the male-female pairs the number of adult progeny was counted. The statistical analysis mirrored that for the longevity assay (Eqn. 2) with the addition of a fixed effect of block. In all experimental populations, the distribution of fitness was non-normal because certain male-female pairs failed to produce offspring. Because this fraction was small (5.3% of pairs set-up overall) and the exact cause is unknown (but may include infertile individuals, refusals to mate, and experimenter error), the analysis was restricted to pairs producing offspring. Results do not change qualitatively if these individuals are included and a non-parametric analysis is performed.

### Water balance and CHC profiles

#### Desiccation tolerance

Survival times under acute desiccation stress of flies from generation 15(10) were measured following the method of Gibbs *et al.* [16]. Briefly, 9 g of desiccant (Drierite®, W. A. Hammond DRIERITE Co. LTD., Xenia, USA) was placed in the bottom of a 30 mL vial and separated from a group of ten one day-old same sex flies by a 1.75 cm porous foam plug. Parafilm® was then placed over the open end of the vial to create an airtight seal. Food was absent from the vials as it may provide a source of water. The resulting nutritional stress is likely mild however compared to strong mortality selection induced by severe desiccation (e.g., see [17]).

Ten replicate vials per sex and population were set up and all were assayed simultaneously. Flies were assessed visually every 30 min for death until all individuals succumbed. From these individual time-to-death data, the best fit mortality model was determined for each population separately by sex using WinModest 1.0.2 as previously described. Female mortality was best described by a Gompertz model, whereas male mortality best fit a logistic model. The underlying mortality parameters of these models (*α* and *β* in females; *α*, *β* and *s* in males) were then compared separately between treatments using nested mixed model ANOVAs as previously described.

#### Rate of water loss, body mass and routine metabolic rate

At generation 29(24), seven replicates groups of 20 same sex individuals from each population were assayed for H_2_O and CO_2_ production using flow-through respirometry. Each group was held within a 10 mL glass chamber covered with an opaque cover, and seven groups were assayed simultaneously along with one empty reference chamber. The seven groups that were assayed together were randomly chosen from the pool of 168 replicates (12 populations×7 replicates×2 sexes), yielding 24 sets. Measurements were conducted at 25°C over a period of three days on 2–4 day-old flies. A FlowBar-8 (Sable Systems) was placed upstream of the chambers to provide them with 50 mL/min of ambient air. A Multiplexer v3 (Sable Systems) was connected down-stream of the chambers to divert the flow from one of the experimental chambers containing flies, and the reference chamber that lacked flies, to a LI-7000 CO_2_/H_2_O analyzer (LI-COR Biosciences, Lincoln, USA) that recorded CO_2_ and H_2_O levels.

Preliminary observations of groups of flies in the respirometry chambers did not reveal any differences in behaviour (see also the results of the locomotor activity assay below). Under these conditions, groups of flies yielded steady state rates of CO_2_ production with no distinguishable CO_2_ peaks; measurements using groups of varying size (i.e. 10, 20 and 30 flies) also showed proportional changes in CO_2_ production rates. Using these steady state rates, metabolic rate and rate of water loss were estimated as the difference between the experimental and reference chambers, accounting for room air CO_2_ variation, and calculated following [37] assuming a respiratory quotient (RQ) of one. To account for drift in the calibration of the LI-7000, baseline data were recorded before each experimental measurement. Measurements were 15 min in length and were taken sequentially from each of the seven experimental chambers. Subsequent to their measurement, each sample of 20 flies was anesthetised with CO_2_ and then weighed as a group to the nearest µg on a MX5 Microbalance (Mettler Toledo, Columbus, USA). H_2_O and CO_2_ emission rates were recorded in Pa were converted in µl/h/mg [38]. The analysis of these rates used a partly nested split-plot design [35]:
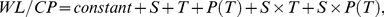
(3)in which *WL/CP* is the average rate of water loss/CO_2_ production (µl/h/mg) of the seven groups of 20 flies of a given sex (*S*) from population (*P*) nested within treatment (*T*). Sex, treatment and their interaction were fixed effects, while population and its interaction with sex were random effects. The F ratio for the treatment effect used the mean square of population nested within treatment as the denominator, and the sex effect used the mean square of sex×population(treatment) as the denominator [35].

#### Body weight

At generation 15(10), the wet weight of 100 male and 100 female 3 day-old adult flies was individually measured to the nearest µg using a MX5 Mettler Toledo (Columbus, OH, USA) microbalance. These individuals were raised in density-controlled vials (100–110 eggs per vial) and adults were starved for eight hours prior to weighing by transferring them vials containing 5 ml standard medium but lacking any live yeast on top. The analysis of these data used a partly nested split-plot design as described above for water loss and routine metabolic rate.

#### CHC assay

At generation 33(28), CHCs were extracted separately from ten individuals per population and sex (all three day-old virgins) by washing individual flies in 100 µL of hexane for approximately 3 min and then vortexing for 1 min. Individual CHCs samples were analyzed using a dual-channel Agilent 6890N fast gas chromatograph fitted with HP-5 phenylmethylsiloxane columns of 30 m×250 µm internal diameter (0.1 µm film thickness), pulsed splitless inlets (at 275°C), and flame ionization detectors (at 310°C). The temperature program began by holding at 140°C for 0.55 min, ramping at 120°C/min to 190°C, slowing to 7°C/min to 260°C, then ramping at 120°C/min to 310°C and holding for 3.5 min. Individual CHC profiles were determined by integration of the area under 38 peaks in females and 28 peaks in males, representing all those that could be consistently identified in all individuals of that sex. The pattern of peaks was broadly consistent with those identified in two previous studies [39], [40] using different populations of *D. melanogaster* and CHCs were labelled with reference to these studies (Table S1; Fig. S3). Three peaks in females (26, 27 and 28; Fig. S3) were not present in either past study and are therefore numbered in order of retention time herein.

Relative proportions of individual CHCs were calculated by dividing the area under each peak by the total area under all peaks for that individual. This corrects for non-biological sources of variation among samples in total CHC concentration that arise from their extraction and subsequent chromatography. Because such technical error can be large even with the use of internal standards [41], [42], we refrain from analyzing total CHC content as a trait itself to permit its use as a control for this error. Because the identities of several CHCs are not shared between the sexes, analyses were performed separately by sex using a mixed model in which population was a random effect nested within treatment. The ideal analysis would have been a multivariate version of this model that simultaneously considered the relative concentration of all CHCs present in a given sex. However, this model could not be fit due to limiting degrees of freedom resulting from the large number of traits compared to the modest number of replicate populations. We therefore performed univariate analyses on each proportionate CHC separately. The F ratio for the treatment effect was constructed using the mean square of population nested within treatment as the denominator. FDR correction was used, with α = 0.05, to account for multiple comparisons [37]. An alternative approach would involve the analysis of a smaller number of principal components of the total variation in CHCs. While reducing the problem of multiple comparisons, the biological interpretation of principal components involving 28 or 38 traits can be challenging.

### Metabolism and activity

#### Locomotor activity assay

At generation 49(44), activity (i.e. number of individual movements) over a 46 h period was measured separately by sex on two day-old males and four day-old females. Flies were placed individually in polycarbonate tubes (5 mm diameter×65 mm long) that contained 0.8 mL of food at one end, capped with a rubber stopper, with the opposite end plugged with cotton. 96 replicate tubes were set up using eight separate individuals from each of the 12 populations and populations and were randomly placed in three DAM2 (Trikinetics, Waltham, MA, USA) activity monitors (32 tubes per monitor). Within each monitor, a single infrared beam bisects each tube to detect motion as the fly moves back and forth. These monitors were placed within an incubator (25°C, 50% relative humidity, 12L∶12D photoperiod) and connected to a personal computer running the DAMSystem303X software (Trikinetics, Waltham, MA, USA) to acquire the activity data from the chambers. While measuring activity in females, daylight savings time ended causing repeat measurements for the period between 1:00 am to 2:00 am (occurring at hour 36 of the assay). These repeat measurements were excluded from the analysis. A spike (<2 h) in activity was also observed when the flies were first introduced into their chambers and, to avoid inclusion of this disturbance effect, the first 2 h of activity data were discarded. The resulting activity data were analyzed using a full-factorial repeated measures ANOVA:
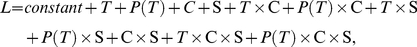
(4)Where *L* is the average locomotor activity over a 10 min period for the eight replicate individuals of sex (*S*) from population (*P*), nested within treatment (*T*), and at time (*C*). Treatment was a between-subject factor while sex and time were within-subject factors. The assumption of sphericity of (co)variances is required for univariate tests of the within-subjects factors and their interactions, and this assumption is unlikely to hold when subjects are measured through time [35]. While a multivariate model is not subject to these concerns, such a model could not be fit due to limiting degrees of freedom. Tests of these factors therefore employed Greenhouse-Geisser epsilon-adjusted significance levels [43]. F ratio tests for treatment, time and sex used the mean square of population(treatment), population(treatment)×time, and population(treatment)×sex as their respective denominators [35]. As with all unreplicated versions of this model, there is no test of population(treatment) or its interaction with any other term because the three-way interaction cannot be estimated separately from the residual.

#### Mitochondrial citrate synthase activity

At generation 39(34), CS activity was measured according to Pichaud *et al.* [44] with minor modifications. Groups of day-old males, ranging from 25–32 individuals per population so as to obtain 20–25 mg of tissue, were collected using light CO_2_ anaesthesia and frozen at −80°C. After 20 days, samples were processed, on ice unless otherwise indicated, by first finely cutting them into progressively smaller pieces for 2 min using dissection scissors. A 19∶1 ratio v/w of homogenization buffer (25 mMTris-HCl, 2 mM EDTA, Triton X-100 0.5% v/v, pH 7.4 at 4°C) was then added and the tissues were homogenized using a PT-DA1307 homogenization generator (Kinematica, Lucerne, Switzerland) using three 10 s pulses with 30 s between each pulse. Samples were then sonicated with a multi-tip VC-505 sonication probe (Sonics & Materials Inc., Newtown, CT, USA) at low intensity for a single 5 s pulse. Homogenized samples were centrifuged at 5500 *g* for 10 min at 4°C and the resulting supernatant was diluted 100× and used to perform the assays.

In a 96 well plate, diluted sample was combined with reaction solution with a final concentration of 50 mMTris-HCl, 0.5 mM oxaloacetate, 0.1 mM5,5′ dithiobis-2-nitrobenzoic acid (DTNB), pH 8.0 at 25°C. Samples were read at 412 nm with a Synergy 2 Multi-Mode Microplate Reader (Biotek, Winooski, VT, USA) before and after the addition of acetyl-CoA (0.3 mM). Assays were performed in triplicate. Control rates without substrate were determined for each assay by subtracting ΔA412/min before and after the addition of acetyl-CoA [45], however values were zero in all cases. The CS reaction was monitored using DTNB at 412 nm using the mM extinction coefficient (ε = 13.6) [46]. All chemicals were from Sigma Chemical Company (Oakville, ON, Canada). The statistical analysis used a nested mixed model in which population was a random effect nested within the fixed effect of treatment. The F ratio for the treatment effect was constructed using the mean square of population nested within treatment as the denominator.

## Supporting Information

Figure S1 **Mortality in 100% oxygen as measured at generation 15(10 hyperoxia).** Male (solid) and female (dashed) *D. melanogaster* evolved under normoxic (black), hypoxic (blue) or hyperoxic (red) conditions and were then raised in a common normoxic environment for two generations prior to conducting the assay. Treatment means of the replicate populations are shown for clarity. There were no significant differences among treatments for any of the parameters underlying these mortality functions in males or females (Table 1). Longevity was measured for 240 individuals/sex/treatment (60 individuals/sex/population), as described in the Methods.
(TIF)

Figure S2 **Mortality under prolonged desiccation stress as measured at generation 15(10 hyperoxia).** Male (solid) and female (dashed) *D. melanogaster* evolved under normoxic (black), hypoxic (blue) or hyperoxic (red) conditions and were then raised in a common normoxic environment for two generations prior to conducting the assay. Treatment means of replicate populations are shown for clarity. There were no significant differences between treatments for any of the parameters underlying these mortality functions in either sex (Table 3). Longevity was measured for 400 individuals/sex/treatment (100 individuals/sex/population), as described in the Methods.
(TIF)

Figure S3 **Mirrored gas chromatographic traces showing the cuticular hydrocarbons of male (red) and female (blue)** ***D. melanogaster*** **.** Individual CHCs that were integrated are sequentially numbered within the profiles of each sex. Unlabelled peaks were not consistently present in all individuals of that sex and were not integrated.
(TIF)

Table S1 **Treatment effects on male and female cuticular hydrocarbons (CHCs) at generation 33(28 hyperoxia).**
(DOC)

## References

[1] Bickler PE, Buck LT (2007). Hypoxia tolerance in reptiles, amphibians, and fishes: Life with variable oxygen availability.. Annu Rev Physiol.

[2] Hoppeler H, Vogt M, Weibel ER, Fluck M (2003). Response of skeletal muscle mitochondria to hypoxia.. Exp Physiol.

[3] Coquin L, Feala JD, McCulloch AD, Paternostro G (2008). Metabolomic and flux-balance analysis of age-related decline of hypoxia tolerance in *Drosophila* muscle tissue.. Mol Syst Biol.

[4] Sohal RS, Agarwal S, Dubey A, Orr WC (1993). Protein oxidative damage is associated with life expectancy of houseflies.. P Natl Acad Sci USA.

[5] Sohal RS (2002). Oxidative stress hypothesis of aging.. Free Radical Bio Med.

[6] Walker DW, Benzer S (2004). Mitochondrial “swirls” induced by oxygen stress and in the *Drosophila* mutant hyperswirl.. P Natl Acad Sci USA.

[7] Frazier MR, Woods HA, Harrison JF (2001). Interactive effects of rearing temperature and oxygen on the development of *Drosophila melanogaster*.. Physiol Biochem Zool.

[8] Henry JR, Harrison JF (2004). Plastic and evolved responses of larval tracheae and mass to varying atmospheric oxygen content in *Drosophila melanogaster*.. J Exp Biol.

[9] Van Voorhies WA (2009). Metabolic function in *Drosophila melanogaster* in response to hypoxia and pure oxygen.. J Exp Biol.

[10] Zhao HW, Zhou D, Nizet V, Haddad GG (2010). Experimental selection for *Drosophila* survival in extremely high O_2_ environments.. PloS One.

[11] Zhou D, Xue J, Chen JM, Morcillo P, Lambert JD (2007). Experimental selection for *Drosophila* survival in extremely low O_2_ environment.. PloS One.

[12] Miwa S, St-Pierre J, Partridge L, Brand MD (2003). Superoxide and hydrogen peroxide production by *Drosophila mitochondria*.. Free Radical Bio Med.

[13] Lighton JRB, Schilman PE, Holway DA (2004). The hyperoxic switch: assessing respiratory water loss rates in tracheate arthropods with continuous gas exchange.. J Exp Biol.

[14] Rascón B, Harrison JF (2010). Lifespan and oxidative stress show a non-linear response to atmospheric oxygen in *Drosophila*.. J Exp Biol.

[15] Klok CJ, Kaiser A, Lighton JRB, Harrison JF (2010). Critical oxygen partial pressures and maximal tracheal conductances for *Drosophila melanogaster* reared for multiple generations in hypoxia or hyperoxia.. J Insect Physiol.

[16] Gibbs AG, Chippindale AK, Rose MR (1997). Physiological mechanisms of evolved desiccation resistance in *Drosophila melanogaster*.. J Exp Biol.

[17] Kwan L, Rundle HD (2010). Adaptation to desiccation fails to generate pre- and postmating isolation in replicate *Drosophila melanogaster* laboratory populations.. Evolution.

[18] Pletcher SD (1999). Model fitting and hypothesis testing for age-specific mortality data.. J Evolution Biol.

[19] Bradley TJ, Briscoe AD, Brady SG, Contreras HL, Danforth BN (2009). Episodes in insect evolution.. Integr Comp Biol.

[20] Jarecki J, Johnson E, Krasnow MA (1999). Oxygen regulation of airway branching in *Drosophila* is mediated by branchless FGF.. Cell.

[21] Rundle HD, Odeen A, Mooers AO (2007). An experimental test for indirect benefits in *Drosophila melanogaster*.. BMC Evol Biol.

[22] Brommer JE, Gustafsson L, Pietiainen H, Merila J (2004). Single-generation estimates of individual fitness as proxies for long-term genetic contribution.. Am Nat.

[23] Partridge L (1980). Mate choice increases a component of offspring fitness in fruit flies.. Nature.

[24] Keller LF, Waller DM (2002). Inbreeding effects in wild populations.. Trends Ecol Evol.

[25] Sheafor BA (2003). Metabolic enzyme activities across an altitudinal gradient: an examination of pikas (genus Ochotona).. J Exp Biol.

[26] Esteva S, Panisello P, Torrella JR, Pages T, Viscor G (2009). Enzyme activity and myoglobin concentration in rat myocardium and skeletal muscles after passive intermittent simulated altitude exposure.. J Sport Sci.

[27] Fedde MR, Faraci FM, Kilgore DL, Cardinet GH, Chatterjee A, Gilles R (1985). Cardiopulmonary adaptations in birds for exercise at high altitude.. Circulation, Respiration, and Metabolism.

[28] Hochachka PW, Stanley C, Merkt J, Sumarkalinowski J (1983). Metabolic meaning of elevated levels of oxidative enzymes in high-altitude adapted animal: an interpretive hypothesis.. Resp Physiol.

[29] Hammond KA, Chappell MA, Cardullo RA, Lin RS, Johnsen TS (2000). The mechanistic basis of aerobic performance variation in red junglefowl.. J Exp Biol.

[30] MacLellan K, Whitlock MC, Rundle HD (2009). Sexual selection against deleterious mutations via variable male search success.. Biol Lett.

[31] Nilson TL, Sinclair BJ, Roberts SP (2006). The effects of carbon dioxide anesthesia and anoxia on rapid cold-hardening and chill coma recovery in *Drosophila melanogaster*.. J Insect Physiol.

[32] Endler JA (1986). Natural selection in the wild.

[33] Schluter D, Nagel LM (1995). Parallel speciation by natural selection.. Am Nat.

[34] Dawson-Scully K, Bukvic D, Chakaborty-Chatterjee M, Ferreira R, Milton SL (2010). Controlling anoxic tolerance in adult *Drosophila* via the cGMP-PKG pathway.. Journal of Experimental Biology.

[35] Quinn G, Keough MJ (2006). Experimental design and data analysis for biologists.

[36] Mockett RJ, Sohal RS, Orr WC (1999). Overexpression of glutathione reductase extends survival in transgenic *Drosophila melanogaster* under hyperoxia but not normoxia.. Faseb Journal.

[37] Benjamini Y, Yekutieli D (2001). The control of the false discovery rate in multiple testing under dependency.. Ann Stat.

[38] Lighton JRB (2008). Measuring metabolic rates: a manual for scientists.

[39] Foley B, Chenoweth SF, Nuzhdin SV, Blows MW (2007). Natural genetic variation in cuticular hydrocarbon expression in male and female *Drosophila melanogaster*.. Genetics.

[40] Everaerts C, Farine JP, Cobb M, Ferveur JF (2010). *Drosophila* cuticular hydrocarbons revisited: mating status alters cuticular profiles.. PloS One.

[41] Blows MW, Allan RA (1998). Levels of mate recognition within and between two *Drosophila* species and their hybrids.. Am Nat.

[42] Savarit F, Ferveur JF (2002). Temperature affects the ontogeny of sexually dimorphic cuticular hydrocarbons in *Drosophila melanogaster*.. J Exp Biol.

[43] SAS Institute I (2008). SAS/STAT® 9.2 User's Guide.

[44] Pichaud N, Chatelain EH, Ballard JWO, Tanguay R, Morrow G (2010). Thermal sensitivity of mitochondrial metabolism in two distinct mitotypes of *Drosophila simulans*: evaluation of mitochondrial plasticity.. J Exp Biol.

[45] Robinson JBJ, Brent LG, Sumegi B, Srere PA, Darley-Usmar VM, Rickwood D, Wilson MT (1987). An enzymatic approach to the study of the Krebs tricarboxylic acid cycle.. Mitochondria: A Practical Approach.

[46] Darveau CA, Hochachka PW, Roubik DW, Suarez RK (2005). Allometric scaling of flight energetics in orchid bees: evolution of flux capacities and flux rates.. J Exp Biol.

