# Supplementary figures and images for: Evolutionary Consequences of Altered Atmospheric Oxygen in Drosophila melanogaster
Source: PLoS One. 2011 Oct 28;6(10):e26876. doi: 10.1371/journal.pone.0026876 (PMC3203924; doi:10.1371/journal.pone.0026876)

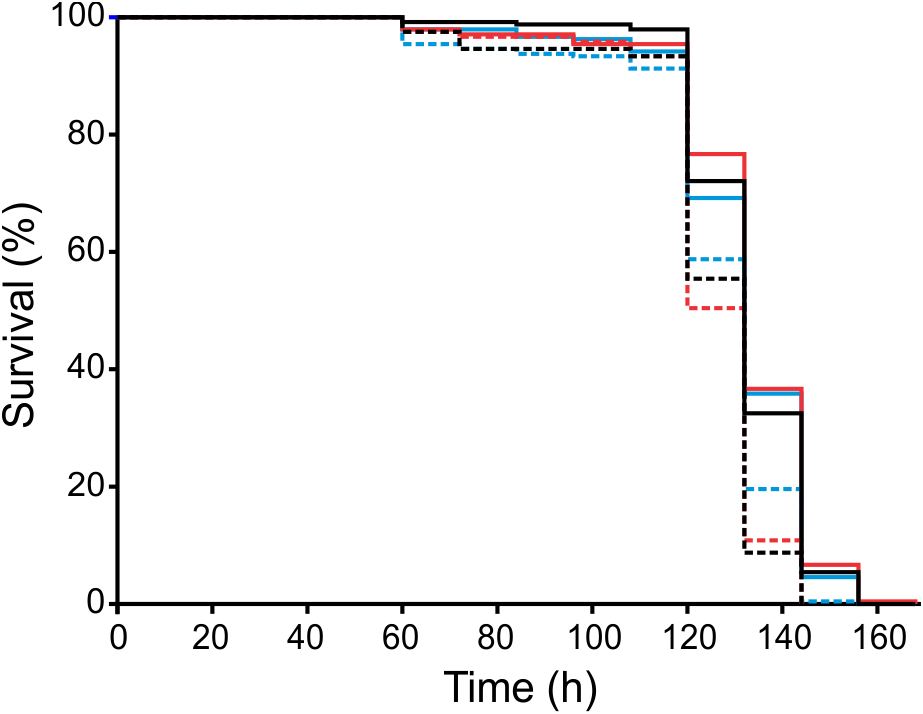

Supplement: Figure S1 — Mortality in 100% oxygen as measured at generation 15(10 hyperoxia). Male (solid) and female (dashed) D. melanogaster evolved under normoxic (black), hypoxic (blue) or hyperoxic (red) conditions and were then raised in a common normoxic environment for two generations prior to conducting the assay. Treatment means of the replicate populations are shown for clarity. There were no significant differences among treatments for any of the parameters underlying these mortality functions in males or females (Table 1). Longevity was measured for 240 individuals/sex/treatment (60 individuals/sex/population), as described in the Methods. (TIF) [file pone.0026876.s001.tif]

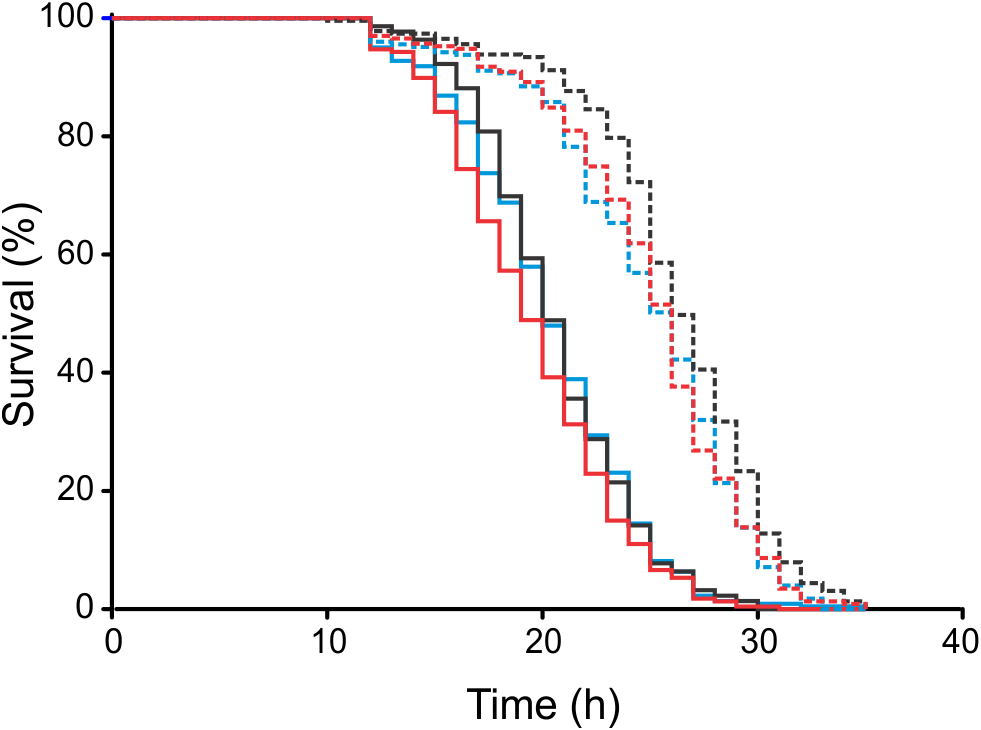

Supplement: Figure S2 — Mortality under prolonged desiccation stress as measured at generation 15(10 hyperoxia). Male (solid) and female (dashed) D. melanogaster evolved under normoxic (black), hypoxic (blue) or hyperoxic (red) conditions and were then raised in a common normoxic environment for two generations prior to conducting the assay. Treatment means of replicate populations are shown for clarity. There were no significant differences between treatments for any of the parameters underlying these mortality functions in either sex (Table 3). Longevity was measured for 400 individuals/sex/treatment (100 individuals/sex/population), as described in the Methods. (TIF) [file pone.0026876.s002.tif]

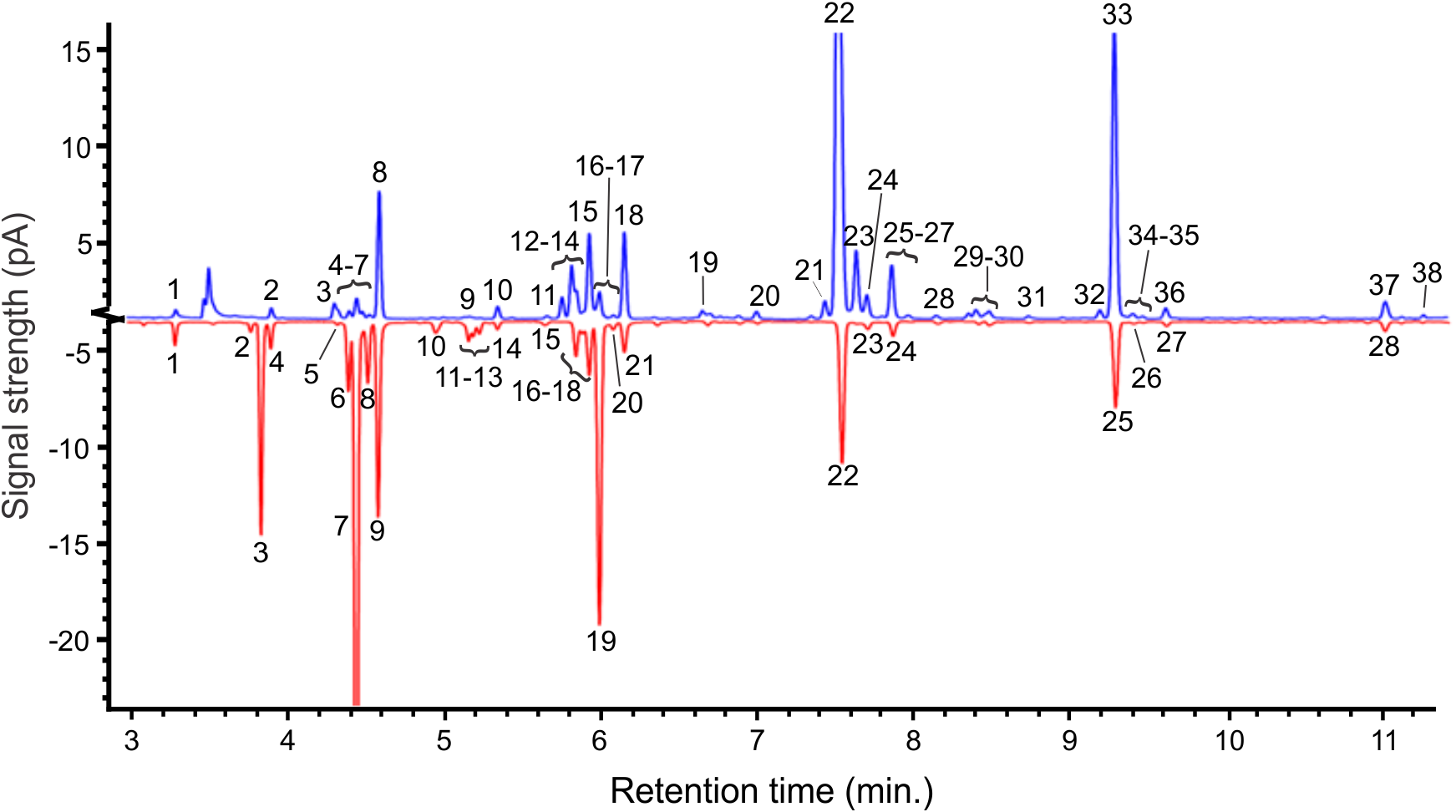

Supplement: Figure S3 — Mirrored gas chromatographic traces showing the cuticular hydrocarbons of male (red) and female (blue) D. melanogaster . Individual CHCs that were integrated are sequentially numbered within the profiles of each sex. Unlabelled peaks were not consistently present in all individuals of that sex and were not integrated. (TIF) [file pone.0026876.s003.tif]
