# Supplementary material for: Evolutionary Consequences of Altered Atmospheric Oxygen in Drosophila melanogaster
Source: PLoS One. 2011 Oct 28;6(10):e26876. doi: 10.1371/journal.pone.0026876 (PMC3203924; doi:10.1371/journal.pone.0026876)
Supplement: Table S1 — Treatment effects on male and female cuticular hydrocarbons (CHCs) at generation 33(28 hyperoxia). (DOC) [file pone.0026876.s004.doc]

**Table S1. Treatment effects on male and female cuticular hydrocarbons (CHCs) at generation 33(28 hyperoxia).Note: Results of separate univariate tests (*d.f.* = 2,9 in all cases), uncorrected for multiple comparisons. Post-hoc comparisons are given for those CHCs that remained significant after a false-discovery rate correction for multiple comparisons (*). Labels correspond to those in Fig. S3**

| **Females** | | | | **Males** | | | | |
| --- | --- | --- | --- | --- | --- | --- | --- | --- |
| **Label** | **Identity** | **F ratio** | **p value** | **Label** | **Identity** | **F ratio** | **p value** | **Post-hoc** |
| 1 | n-Heneicosane | 0.40 | 0.680 | 1 | n-Heneicosane | 1.47 | 0.281 |  |
| 2 | n-Docosane | 0.50 | 0.624 | 2 | (Z)-9-Docosene | 7.37 | 0.013* | 40>21=5 |
| 3 | (Z,Z)-7,11-Tricosadiene | 1.67 | 0.242 | 3 | (Z)-7-Docosene | 3.47 | 0.076 |  |
| 4 | (Z)-9-Tricosene | 5.69 | 0.025 | 4 | n-Docosane | 2.63 | 0.126 |  |
| 5 | (Z)-7-Tricosene | 1.02 | 0.398 | 5 | 2-Methyldocosane | 11.75 | 0.003* | 40>21=5 |
| 6 | (+)-6-Tricosene | 0.19 | 0.831 | 6 | (Z)-9-Tricosene | 2.91 | 0.106 |  |
| 7 | (Z)-5-Tricosene | 0.29 | 0.754 | 7 | (Z)-7-Tricosene | 2.36 | 0.150 |  |
| 8 | n-Tricosane | 2.36 | 0.150 | 8 | (Z)-5-Tricosene | 0.52 | 0.614 |  |
| 9 | (Z,Z)-7,11-Tetracosadiene | 1.29 | 0.322 | 9 | n-Tricosane | 10.19 | 0.005* | 21>5=40 |
| 10 | n-Tetracosane | 0.35 | 0.715 | 10 | 2-Methyltricosane | 0.52 | 0.609 |  |
| 11 | (Z,Z)-9,13-Pentacosadiene | 1.51 | 0.272 | 11 | (Z)-9-Tetracosene | 2.47 | 0.140 |  |
| 12 | (Z,Z)-7,11-Pentacosadiene | 0.21 | 0.815 | 12 | (Z)-7-Tetracosene | 4.73 | 0.040* |  |
| 13 | (x)-12-Pentacosene or Pentacosadiene | 0.77 | 0.490 | 13 | (Z,Z)-5, 9-Tetracosadiene | 2.56 | 0.131 |  |
| 14 | 2-Methyltetracosane | 0.05 | 0.949 | 14 | n-Tetracosane | 18.64 | <0.001* | 21>5>40 |
| 15 | (Z)-9-Pentacosene | 4.44 | 0.046 | 15 | (x)-12-Pentacosene | 1.76 | 0.226 |  |
| 16 | (Z)-7-Pentacosene | 2.52 | 0.135 | 16 | 2-Methyltetracosane | 2.12 | 0.176 |  |
| 17 | (Z)-5-Pentacosene | 3.34 | 0.082 | 17 | (Z,Z)-7,11-Pentacosadiene | 4.08 | 0.055 |  |
| 18 | n-Pentacosane | 0.50 | 0.622 | 18 | (Z)-9-Pentacosene | 10.04 | 0.005* | 5>21=40 |
| 19 | (Z,Z)-7,11-Hexacosadiene | 4.81 | 0.038 | 19 | (Z)-7-Pentacosene | 3.81 | 0.063 |  |
| 20 | n-Hexacosane | 0.58 | 0.580 | 20 | (Z)-5-Pentacosene | 1.57 | 0.259 |  |
| 21 | (Z,Z)-9,13-Heptacosadiene | 0.60 | 0.569 | 21 | n-Pentacosane | 16.20 | 0.001* | 40<21=5 |
| 22 | (Z,Z)-7,11-Heptacosadiene | 3.34 | 0.082 | 22 | 2-Methylhexacosane | 2.87 | 0.108 |  |
| 23 | (Z)-9-Heptacosene | 0.30 | 0.746 | 23 | (Z)-7-Heptacosene | 6.60 | 0.017* | 5>21=40 |
| 24 | (Z)-7-Heptacosene | 4.63 | 0.041 | 24 | n-Heptacosane | 8.47 | 0.009* | 40<21=5 |
| 25 | n-Heptacosane | 2.29 | 0.157 | 25 | 2-Methyloctacosane | 1.51 | 0.273 |  |
| 26 | unidentified | 0.15 | 0.861 | 26 | (Z)-7-Nonacosene | 1.52 | 0.269 |  |
| 27 | unidentified | 8.35 | 0.009 | 27 | n-Nonacosane | 11.20 | 0.004* | 40<21=5 |
| 28 | unidentified | 1.55 | 0.264 | 28 | 2-Methyltriacontane | 8.40 | 0.009* | 5>21=40 |
| 29 | (Z,Z)-7,11-Octacosadiene | 0.72 | 0.511 |  |  |  |  |  |
| 30 | 2-Methylheptacosane | 1.32 | 0.313 |  |  |  |  |  |
| 31 | n-Octacosane | 0.63 | 0.553 |  |  |  |  |  |
| 32 | (Z,Z)-9,13-Nonacosadiene | 0.11 | 0.895 |  |  |  |  |  |
| 33 | (Z,Z)-7,11-Nonacosadiene | 1.08 | 0.381 |  |  |  |  |  |
| 34 | 2-Methyloctacosane | 0.48 | 0.632 |  |  |  |  |  |
| 35 | (Z)-7-Nonacosene | 4.35 | 0.048 |  |  |  |  |  |
| 36 | n-Nonacosane | 1.07 | 0.382 |  |  |  |  |  |
| 37 | 2-Methyltriacontane | 0.43 | 0.665 |  |  |  |  |  |
| 38 | n-Hentriacontane | 10.77 | 0.004 |  |  |  |  |  |
